# Long-term results following atrioventricular septal defect repair

**DOI:** 10.1186/s13019-023-02355-6

**Published:** 2023-08-23

**Authors:** Katja Schumacher, Mateo Marin Cuartas, Sabine Meier, Muhammed Ikbal Aydin, Michael Andrew Borger, Ingo Dähnert, Martin Kostelka, Marcel Vollroth

**Affiliations:** 1 Department of Cardiac Surgery, University, Leipzig Heart Center, Strümpellstrasse 39, 04289 Leipzig, Germany; 2 Department of Pediatric Cardiology, Leipzig Heart Center, Leipzig, Germany

**Keywords:** Atrioventricular septal defect, AVSD, Pediatric cardiac surgery, Complete AV canal, Congenital heart disease, Freedom from reoperation, Long-term results

## Abstract

**Background:**

Atrioventricular septal defects (AVSD) represent 4–7% of congenital cardiac malformations. Definitive early repair is favored over prior pulmonary artery banding and delayed definitive repair in many centers. The aim of this study was to analyze long-term outcomes following AVSD repair over a 21-year period.

**Methods:**

A total of 202 consecutive patients underwent surgical AVSD correction between June 1999 and December 2020. Surgery was performed using the double-patch technique. The study data were prospectively collected and retrospectively analyzed. Primary outcomes were In-hospital mortality and overall long-term freedom from reoperation.

**Results:**

Median age at operation was 120 days (IQR 94–150), median weight was 5.0 kg (4.2–5.3). None of the patients died within the first 30 postoperative days. In-hospital mortality was 0.5% (1/202 patients). Median follow-up was 57 months (11–121). Overall freedom from reoperation at 5, 10 and 15 years was 91.8%, 86.9% and 86.9%, respectively.

**Conclusion:**

AVSD repair with the double-patch technique is a safe and effective procedure with good early postoperative outcomes and low long-term reoperation rates.

## Introduction

Atrioventricular septal defects (AVSD) represent 4–7% of all congenital cardiac malformations and are commonly associated with Down syndrome [1]. Surgical management changed over the last decades from a two-stage approach (i.e., initial pulmonary artery banding (PAB) followed by a late definitive repair) towards an early corrective surgery strategy at the age of 3–6 months [2]. Due to improvements in the surgical technique, peri- and postoperative management as well as diagnostic imaging, early mortality rates following AVSD repair decreased from up to 73% in the 1950s and 1960s [3] to 2.5% in the current era [4]. However, despite improved survival, residual atrioventricular valve regurgitation requiring reoperation remains a major problem. Reoperation rates up to 15% have been reported [5–7]. Besides atrioventricular (AV) valve dysfunction, left ventricular outflow tract obstruction (LVOTO) has been described as a common complication following surgical repair of AVSD occurring in 2–7% of the patients [8]. Furthermore, there are still controversial opinions about the ideal age to perform surgery and if palliative interventions like PAB still play a role in patients younger than 3 months [9, 10]. Given the above described gaps in the evidence and the lack of long-term follow-up, we performed this study to analyze the postoperative outcomes, especially reoperation rates due to AV valve dysfunction and LVOTO, following early definitive AVSD repair with the double-patch technique during a total experience of 21 years at Leipzig Heart Center.

## Patients and methods

### Ethical statement

This research project was approved by the University of Leipzig. Individual patient informed consent was waived due to the anonymous data management and the retrospective nature of this study.

### Study cohort

A total of 202 consecutive patients with total AVSD undergoing definitive repair between June 1999 and December 2020 were included in our analysis. Data were prospectively collected in the institutional database and retrospectively analyzed. Patients with unbalanced ventricles as well as intermediate or partial AVSD were excluded from the analysis.

### Surgical technique

During the 21-year study period, four cardiac surgeons performed AVSD repair with minimal variation in surgical technique or perioperative management [7]. Surgery was performed on cardiopulmonary bypass with aortic and bicaval cannulation in moderate hypothermia (rectal temperature 28 °C). Cardioplegic arrest was achieved using St. Thomas Hospital cardioplegia solution (35 ml/kg body weight, repeated application of 25 ml/kg body weight after 90 min cardioplegic arrest). The surgical reconstruction was performed using a double-patch technique in all patients.

**Fig. 1:**
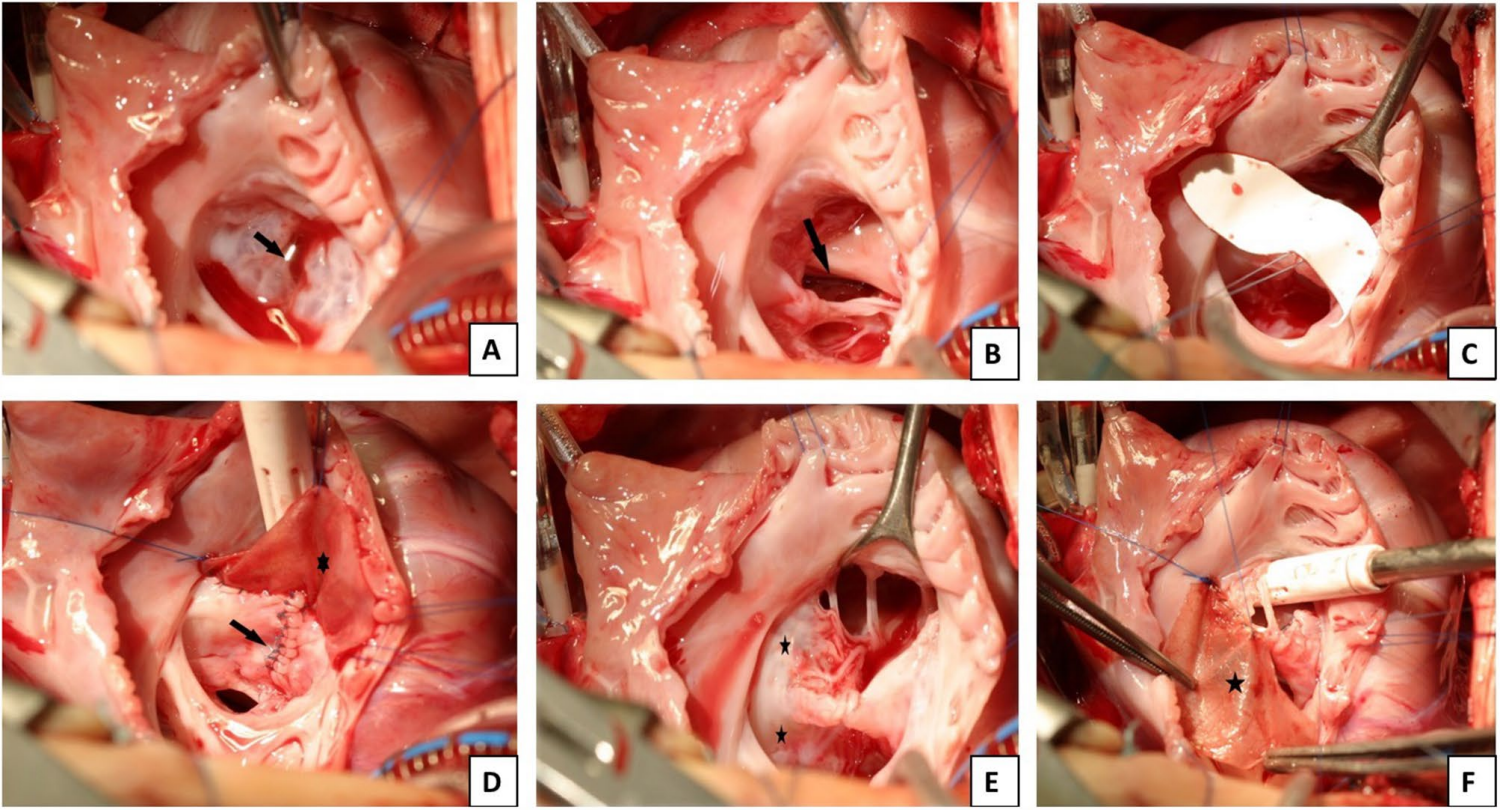
Surgical technique: intraoperative pictures. **(A)** Sealing test. The arrow marks the cleft. **(B)** The arrow shows the large VSD.  **(C)** Ventricular septal defect closure with GoreTex patch. **(D)** Cleft and ASD closure. The cleft was closed with 6 − 0 interuppted Cardionyl sutures (arrow). **(E)** Common valve. The superior leaflets are marked with stars. **(F)** The ASD was closed with autologous pericardial patch using running 5 − 0 Prolene sutures (star)

All operations were performed through median sternotomy. After aortic and bicaval cannulation, valve exposure was obtained through right atriotomy parallel to the AV groove to inspect the anatomy of the valve. Cold saline solution was used to identify the line of coaptation of the common AV valve and the Rastelli classification (Fig. 1, A). A retractor was used to inspect the right atrioventricular valve components and the subvalvular apparatus. The size of the VSD was evaluated (Fig. 1, B). The ventricular septal defect (VSD) was closed by sewing in a Gore-Tex patch (W L Gore, Flagstaff, AZ) onto the right side of the intraventricular septum with continuous double armed 5 − 0 Prolene suture (Johnson and Johnson, New Brunswick, NJ) (Fig. 1, C). The cleft is closed with interrupted sutures of Cardionyl 6 − 0 (Fig. 1, D). Closure of the atrial septal defect (ASD) was performed with a glutaraldehyde-pretreated autologous pericardial patch. The patch was fixed with a running suture (Prolene 5 − 0) to the crest where superior and inferior bridging leaflets are attached to the VSD patch (Fig. 1, F).

After weaning off cardiopulmonary bypass, epicardial or transesophageal echocardiography was performed to evaluate and document the surgical results.

### Follow-up

Patients’ demographics and surgical data were obtained from the medical records of the Leipzig Heart Center. Follow-up data were collected during a subsequent visit in our outpatient clinic or by referring pediatric cardiologists. The median follow-up was 49 months (IQR 8-114). In-hospital mortality and early reoperation were defined as death or reoperation before hospital discharge. The degree of atrioventricular valve regurgitation was subjectively classified as mild, moderate, or severe based on the width of the color Doppler jet.

### Statistical methods

Categorical variables are expressed as frequencies and percentages throughout the manuscript. Continuous variables are expressed as mean ± standard deviation for normally distributed variables and median and interquartile range (IQR, 25% IQ – 75% IQ) for non-normally distributed variables. Freedom from reoperation was estimated using the Kaplan–Meier method. Statistical analyses were performed using the SPSS software package, version 25.0 (IBM Corp, Armonk, NY, USA) and Microsoft Excel 2019 for Mac. In order to determine clinical predictors of long-term reoperation, univariate Cox proportional hazards regression models were performed. Perioperative variables that had a univariable value of *P* < 0.5 or those judged to be clinically important were submitted to a multivariable Cox proportional hazard model by backward stepwise selection. The results of the Cox models are reported as hazard ratios (HR) with 95% confidence interval (CI).

## Results

### Patient baseline characteristics

Baseline characteristics are shown in Table 1. The Rastelli classification was determined based on echocardiographic and surgical reports [Rastelli A: n = 118 (58.4%), Rastelli B: n = 29 (14.4%), Rastelli C: n = 55 (27.2%)]. Median age at operation was 120 days (94–150) and median weight was 5.0 kg (4.2–5.3). A total of 42 (20.8%) patients were younger than 3 months and 149 (73.8%) patients had Down syndrome. Secundum atrial septal defect (n = 144, 71.3%) and patent foramen ovale (n = 34, 16.8%) were the most common associated congenital heart defects. A total of 17 (8.4%) patients underwent previous cardiac surgery prior to AVSD repair. Table 1 depicts further information on major concomitant cardiac anomalies.

**Table 1:** Baseline characteristics

Variable	n = 202
Age (days) - median (IQR)	120 (94 - 150)
Age category	
0 - 30 days - n (%)	2 (0.9)
1 - 3 months - n (%)	40 (19.8)
4 - 6 months - n (%)	128 (63.4)
7 - 12 months - n (%)	22 (10.9)
1 - 18 years - n (%)	10 (5.0)
Weight (kg) - median (IQR)	5.0 (4.2 - 5.3)
Female - n (%)	112 (55.4)
Previous pulmonary artery banding - n (%)	7 (3.5)
Previous cardiac surgery - n (%)	17 (8.4)
Rastelli A - n (%)	118 (58.4)
Rastelli B - n (%)	29 (14.4)
Rastelli C - n (%)	55 (27.2)
Down syndrome - n (%)	149 (73.8)
Tetralogy of Fallot - n (%)	11 (5.4)
Atrial septal defect type II - n (%)	144 (71.3)
Patent ductus arteriosus	46 (22.8)
Patent foramen ovale	34 (16.8)
Aortic anomalies	11 (5.4)

IQR interquartile range

### Intraoperative variables

Intraoperative details are presented in Table 2. The median cardiopulmonary bypass time was 115 min (105–138), and the median aortic cross-clamp time was 71 min (64–83). In 200 patients (99.0%), additional procedures were performed (Table 2.)

**Table 2:** Intraoperative details

Variable	n = 202
CPB time (min) − median (IQR)	115 (105 − 138)
Aortic cross-clamp time (min) − median (IQR)	71 (64 − 83)
Additional procedures	200 (99.0)
TOF correction − n (%)	10 (5.0)
ASD Type II closure − n (%)	144 (71.3)
PFO closure − n (%)	34 (16.8)
PDA ligation − n (%)	27 (13.4)
Lowest temperature (Celsius) − median (IQR)	28 (26 − 28)

ASD atrial septal defect; CPB cardiopulmonary bypass time; min minutes; IQR interquartile range; PDA patent ductus arteriosus; PFO patent foramen ovale; TOF Tetralogy of Fallot

### Early postoperative outcomes

None of the patients died within the first 30 postoperative days. The In-hospital mortality was 0.5%. Median intensive care unit (ICU) stay was 8 days [6–14]. A total of 6 (3.0%) patients presented with major postoperative bleeding requiring surgical re-exploration, and 2 (0.9%) patients required postoperative support with veno-arterial extracorporeal membrane oxygenation (VA-ECMO) due to postcardiotomy cardiogenic shock. Permanent pacemaker implantation was performed in 6 (3.0%) patients. The indication for pacemaker implantation was third-degree atrioventricular block in 4 (2.0%) patients, second-degree atrioventricular block in 1 (0.6%) patient, and sinus node dysfunction in 1 (0.5%) patient. Thirty-one (15.3%) patients developed chylothorax postoperatively. Early reoperation was required in 4 (2%) patients due to severe residual left atrioventricular valve (AV) regurgitation in the postoperative echocardiography. Amongst them, left AV redo repair could be performed in 3 (1.5%) patients while 1 (0.5%) patients required mechanical left AV replacement. Left AV regurgitation in the discharge echocardiography was none in 26 (12.8%) patients, mild in 85 (42.1%) patients (including two patients with prosthetic valve replacement without any prosthetic dysfunction), moderate in 90 (44.6%) patients and severe in one case (0.5%). Right AV regurgitation was none in 57 (28.2%) patients, mild in 100 (49.5%) patients, moderate in 45 (22.3%) patients. Major postoperative complications are summarized in Table 3.

**Table 3:** Postoperative outcomes

Variable	n = 202
**Major postoperative complications**	
Chylothorax - n (%)	31 (15.3)
Pacemaker - n (%)	6 (3.0)
ECMO - n (%)	2 (0.9)
Bleeding requiring re-exploration - n (%)	6 (3.0)
**Postoperative outcomes**	
In-hospital mortality (%)	1 (0.5)
ICU stay (days) - median (IQR)	8 (6 - 14)
Hospital stay (days) - median (IQR)	15 (12 - 22)
Residual MR - n (%)	176 (87.1)
Mild - n (%)	85 (42.1)
Moderate - n (%)	90 (44.6)
Severe - n (%)	1 (0.5)
Residual TR - n (%)	145 (71.8)
Mild - n (%)	100 (49.5)
Moderate - n (%)	45 (22.3)

ECMO extracorporeal membrane oxygenation; ICU intensive care unit; IQR interquartile range; MR mitral regurgitation; TR tricuspid regurgitation, VSD ventricular septal defect

### Long-term outcomes

**Fig. 2:**
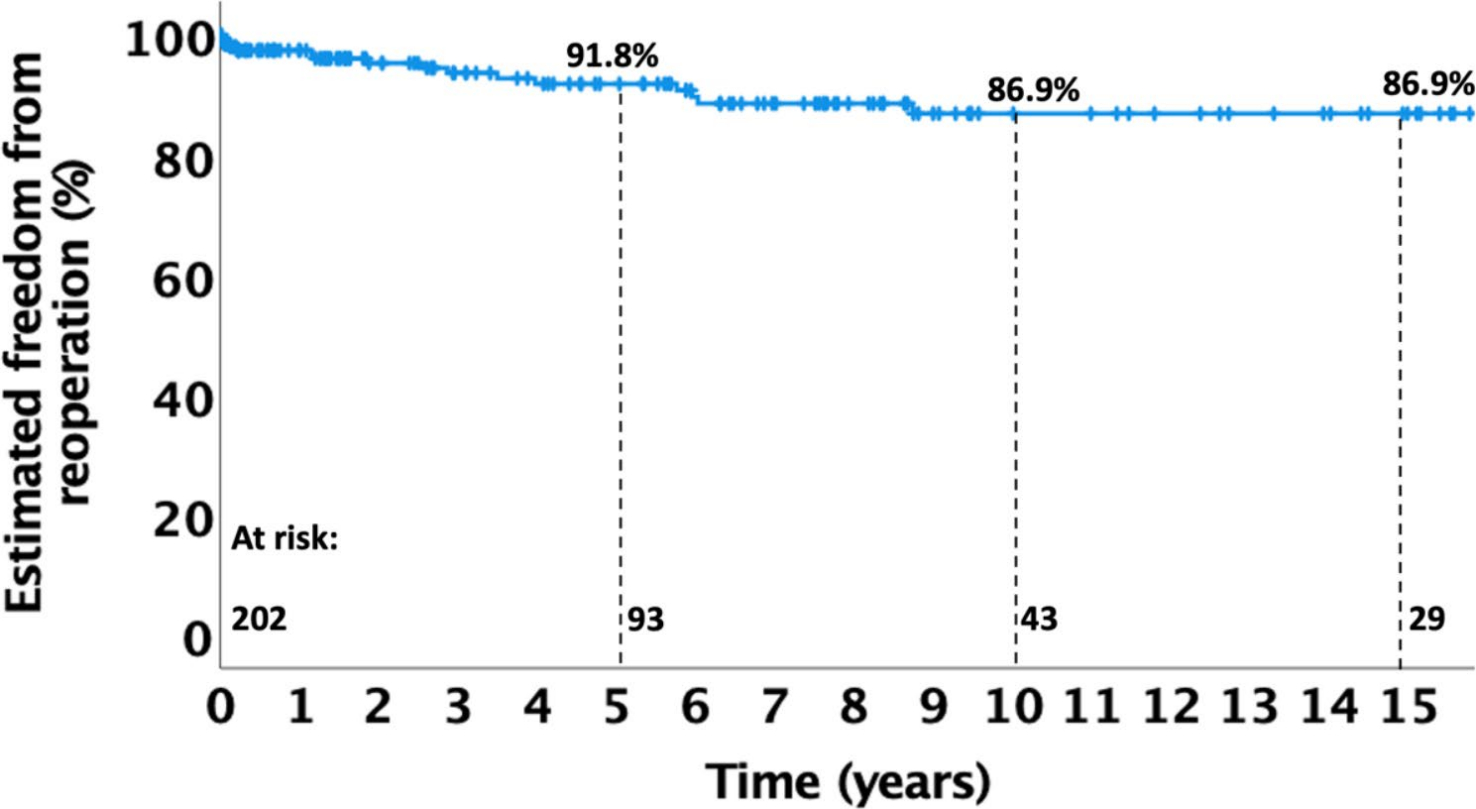
Overall freedom from reoperation

Freedom from overall reoperation at 5, 10 and 15 years was 91.8%, 86.9% and 86.9%, respectively (Fig. 2). The reasons for reoperations are shown in Table 4. There were 8 (4.0%) patients in need for reoperation due to left AV regurgitation, one (0.5%) due to right AV regurgitation, 4 due to subaortic stenosis (2.0%) and one due to residual VSD (0.5%). Two patients (1.0%) received left AV and SAS repair during the same surgery.

**Table 4:** Reasons for reoperation

Variable	n = 202
Left AV valve regurgitation - n (%)	8 (4.0)
Right AV valve regurgitation - n (%)	1 (0.5)
LVOTO - n (%)	4 (2.0)
VSD – n (%)	1 (0.5)
Left AV valve regurgitation + SAS – n (%)	2 (1.0.)

AV atrioventricular; LVOTO left ventricular outflow tract obstruction; VSD ventricular septal defect

## Discussion

The current study represents a single-center experience over a period of 21 years in patients undergoing early definitive AVSD repair with the double-patch technique. The main findings of our study are:

1. AVSD repair can be performed with very low in-hospital mortality.
2. Re-operation rates due to AV valve regurgitation and LVOTO are low.

Surgery of patients with total AVSD was always performed using the double-patch technique whereas many centers prefer the modified single-patch approach. Although there are several publications favoring one technique over the other, there is still no consent in the literature. Modified single-patch technique is described to be associated with significantly shorter cardiopulmonary bypass and crossclamp time as well as shorter hospital stays [11–14]. However, there is no consistent difference in early mortality, overall mortality, residual AV regurgitation, LVOTO, reoperation or postoperative atrioventricular block [11, 14]. To this day, there a no randomized trials comparing single-patch versus double-patch technique. Like our analysis, most of the published studies are retrospective and often single-centre or even single-surgeon series. In conclusion, there is no existing data confirming superiority of one technique over the other.

Our analysis demonstrates that AVSD repair can be performed with low in-hospital mortality as well as a low long-term reoperation rate. However, AV valve regurgitation remains a major problem after AVSD repair affecting morbidity and mortality [5, 15]. Preoperative [16] and early postoperative [3] AV valve regurgitation, incomplete cleft closure, double orifice left AV valve, associated cardiovascular anomalies [5], and the absence of Down syndrome [3, 17, 18] have been identified as risk factors for AV valve dysfunction and reoperation [19]. There are different reasons for residual/recurrent AV valve regurgitation reported in the literature: non- or partial cleft closure, degeneration of valve tissue, annular dilatation, and suture dehiscence between the leaflets and the patch due to congestive heart failure [15]. In our cohort, cleft repair dehiscence was a major cause of residual AV valve regurgitation. There were four patients requiring reoperation due to left AV regurgitation after prior AVSD repair. In two cases, residual cleft dehiscence occurred which was closed during reoperation. In one case, the left AV presented separated from the VSD patch, refixation was performed. The last patient which was in need for left AV replacement after unsuccessful repair had a complex valve anatomy with undifferentiated papillary muscles and anomalous chordae as well as residual central and anterolateral cleft. During repair attempt, cleft closure and anterolateral commissurotomy was performed. However, there was still severe left AV regurgitation afterwards resulting in left AV replacement with a St. Jude 19 mm valve. Repair techniques are preferred over replacement due to an increased mortality risk of up to 50% in addition to valve-related morbidity, including thromboembolic and hemorrhagic complications, as well as the need for future valve replacement due to outgrowth [20, 21].

After reoperation for atrioventricular valve dysfunction, LVOTO is the second most frequent cause for reoperation after AVSD repair [8, 22]. Previously reported anatomical risk factors for LVOTO include fibromuscular obstruction, septal hypertrophy, and valve-related sources of obstruction, as well as the anterolateral muscle bundle of the left ventricle, a feature of the left ventricular outflow tract in all AVSD patients [22, 23]. The complexity of the mechanisms causing LVOTO demands for various surgical repair techniques [22]. In our cohort, a prevalence of reoperation for LVOTO at 3.0% is in line with a previous series reporting a 3.7% reoperation rate [24]. LVOTO repair surgery was performed through median sternotomy on cardiopulmonary bypass in cardiac arrest. After transverse aortotomy, fibrous subaortic tissue was resected. In four cases, there was also performed a myotomy or even myectomy. Afterwards, Hegar dilatators were used to ensure adequate LVOT diameter even before weaning the patient from bypass. Further studies suggested that single patch repair may be associated with LVOTO development compared to double-patch technique [22] although the data is not consistent [25]. However, since the double-patch technique was favored at our institution, we could not assess the impact of the different surgical techniques on the reoperation rate for LVOTO.

In our cohort, 6 (3.0%) of the patients required permanent pacemaker implantation after surgery. In the literature, rates for pacemaker implantation range from 1.4% to up to 11.4% [7, 26–29]. A few groups also report no atrioventricular blocks requiring permanent pacemaker implantation after AVSD repair [12, 30]. Some authors hypothesize a difference in the need for pacemaker implantation between single-patch and double-patch techniques; however, this difference could not be significantly proven [11, 31, 32].

Unfortunately, most likely due to a small number of events of interest we were not able to identify risk factors for perioperative complications, mortality and reoperation. Younger age is often described as risk factor for mortality after AVSD repair [5]. Furthermore, there is also data demonstrating a higher risk for reoperation in younger patients especially in those < 3 months [2, 33]. Children younger than 3 months were also described to present with more severe left AV valve regurgitation at discharge leading to higher risk for reoperation [34]. Concomitant with younger age, lower weight < 4 kg has been described with higher risk for reoperation [33]. The majority of patients in our cohort (n = 128 (63.4%)) was between 4 and 6 months old which is the preferred age at our institution for AVSD repair. In case of severe symptom burden at a younger age, we usually perform pulmonary banding prior to final AVSD repair with good results. Because AVSD frequently goes along with Down syndrome (73.8% in our cohort), the role of trisomy 21 as a risk factor for reoperation and mortality following AVSD repair is analyzed in many studies. Interestingly, the absence of Down syndrome is mainly associated with higher degrees of left AV valve regurgitation after repair [35] leading to higher rates of reoperation [18]. Patients with Down syndrome were described to need less and later reoperations compared to patients without Down syndrome [33]. These correlations could be explained by the fact that AV valve anatomy in patients with trisomy 21 differs from those in patients without trisomy 21. There is often more valve tissue in patients with Down syndrome allowing an easier reconstruction of the valve [33]. Furthermore, there is a higher prevalence of anomalies of the left AV valve as well as LVOT in patients without Down syndrome [17].

### Limitations

There are several limitations of this study that should be noted. First, this is a single‑center retrospective study with the corresponding limitations associated with its nature. Moreover, the study does not compare different treatment strategies. Some parameters, such as the degree of left AV valve regurgitation at discharge, have been subjectively classified and not based on objective quantitative echocardiographic parameters. Finally, we were unable to identify independent predictors of poor outcomes during follow up, most likely due to the small number of events of interest.

## Conclusion

AVSD repair with the double-patch technique is a safe and effective procedure with good early postoperative outcomes and low long-term reoperation rates.

## Data Availability

The data underlying this article will be shared upon request to the corresponding author.
